# The atomic-level structure of bandgap engineered double perovskite alloys Cs_2_AgIn_1−*x*_Fe_*x*_Cl_6_†

**DOI:** 10.1039/d0sc05264g

**Published:** 2020-12-08

**Authors:** Fuxiang Ji, Feng Wang, Libor Kobera, Sabina Abbrent, Jiri Brus, Weihua Ning, Feng Gao

**Affiliations:** Department of Physics, Chemistry and Biology (IFM), Linköping University Linköping SE-581 83 Sweden weihua.ning@liu.se feng.gao@liu.se; Institute of Macromolecular Chemistry of the Czech Academy of Sciences Heyrovskeho nam. 2, 162 06, Prague 6 Czech Republic kobera@imc.cas.cz; Key Laboratory of Flexible Electronics (KLOFE) and Institute of Advanced Materials (IAM), Nanjing Tech University 30 South Puzhu Road Nanjing 211816 P. R. China

## Abstract

Although lead-free halide double perovskites are considered as promising alternatives to lead halide perovskites for optoelectronic applications, state-of-the-art double perovskites are limited by their large bandgap. The doping/alloying strategy, key to bandgap engineering in traditional semiconductors, has also been employed to tune the bandgap of halide double perovskites. However, this strategy has yet to generate new double perovskites with suitable bandgaps for practical applications, partially due to the lack of fundamental understanding of how the doping/alloying affects the atomic-level structure. Here, we take the benchmark double perovskite Cs_2_AgInCl_6_ as an example to reveal the atomic-level structure of double perovskite alloys (DPAs) Cs_2_AgIn_1−*x*_Fe_*x*_Cl_6_ (*x* = 0–1) by employing solid-state nuclear magnetic resonance (ssNMR). The presence of paramagnetic alloying ions (*e.g.* Fe^3+^ in this case) in double perovskites makes it possible to investigate the nuclear relaxation times, providing a straightforward approach to understand the distribution of paramagnetic alloying ions. Our results indicate that paramagnetic Fe^3+^ replaces diamagnetic In^3+^ in the Cs_2_AgInCl_6_ lattice with the formation of [FeCl_6_]^3−^·[AgCl_6_]^5−^ domains, which show different sizes and distribution modes in different alloying ratios. This work provides new insights into the atomic-level structure of bandgap engineered DPAs, which is of critical significance in developing efficient optoelectronic/spintronic devices.

## Abstract 2

Through Fe^3+^-alloying, the bandgap of benchmark double perovskite Cs_2_AgInCl_6_ can be tuned from 2.8 eV to 1.6 eV. The atomic-level structure of Cs_2_AgIn_1−*x*_Fe*_x_*Cl_6_ was revealed by solid-state nuclear magnetic resonance (ssNMR).

## Graphical abstract

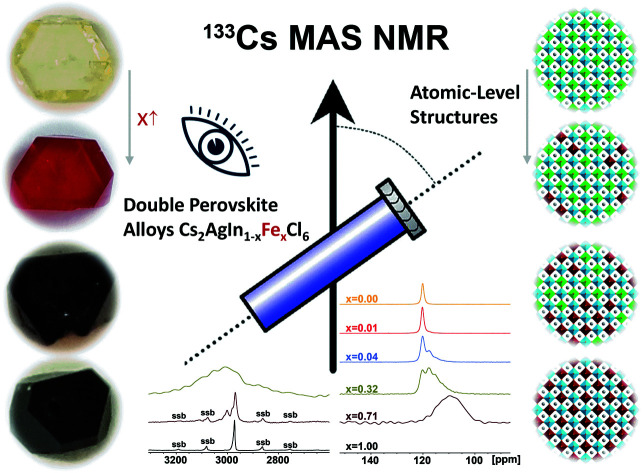

## Introduction

Lead (Pb) halide perovskites have received considerable attention for photovoltaics field.^1–3^ However, the presence of toxic Pb and intrinsic poor stability are the main bottlenecks for their further application.^4^ A promising approach to solve these issues is to replace divalent Pb^2+^ with monovalent B^+^ and trivalent B^3+^ metal ions, forming a double perovskite with the formula of A_2_B^+^B^3+^X_6_ (A = Cs^+^, CH_3_NH_3_^+^; B = metal ions; X = Cl^−^, Br^−^, I^−^).^5–7^ Unfortunately, the large bandgaps of the current double perovskites limit their practical applications.

Alloying/doping is a simple yet efficient method to tune the bandgaps of a wide range of materials, including traditional inorganic semiconductors,^8,9^ oxide-based perovskites,^10^ Pb-based perovskites,^11^ as well as lead-free halide double perovskites.^12–15^ For example, trivalent Sb^3+^-alloying has been employed to decrease the bandgap of Cs_2_AgBiBr_6_ and Cs_2_AgInCl_6_. However, the bandgaps of the alloys in both cases are still large for photovoltaic applications.^12,13^ In addition, multivalent Tl (Tl^+^/Tl^3+^) has also been introduced to decrease the bandgap of Cs_2_AgBiBr_6_ from 2.0 eV to 1.40 eV by Karunadasa and coworkers.^14^ Considering the high toxicity of Tl, they further doped less toxic Sn^2+^ into Cs_2_AgBiBr_6_ crystals, reaching a promising bandgap of 1.48 eV.^15^ However, the oxidatively unstable Sn^2+^ makes the doped perovskites highly sensitive to the ambient atmosphere. Therefore, finding rational alloying ions to tune the bandgaps of benchmark double perovskites remains challenging. During the preparation of this manuscript, Fe^3+^-alloying strategy was reported to reduce the bandgap of double perovskite Cs_2_AgInCl_6_.^21^

In parallel with the material development using the alloying strategy, fundamental understanding of these double perovskite alloys is also of critical importance. However, the atomic-level understanding of various dopants/alloys in halide DPAs is at a very early stage; recently, Michaelis and coworkers provided the first report for the long- to short-range structural elucidation of white-light-emitting DPAs.^16^ In this aspect, solid-state NMR (ssNMR), where very small chemical shifts within a given nucleus type record precisely the local chemical environment of its chemically inequivalent sites,^17–20^ provide a unique and powerful approach to understand the local atomic-level structures of double perovskite alloys.

Herein, we alloy magnetic ions (Fe^3+^-alloying) into the benchmark Cs_2_AgInCl_6_,^21^ and tune the bandgap of Cs_2_AgIn_1−*x*_Fe_*x*_Cl_6_ over a range from 2.8 eV to 1.6 eV. We investigate the atomic-level structure of these double perovskite alloys using ^133^Cs and ^115^In ssNMR spectroscopy, where the paramagnetic Fe^3+^ provides rich information on nuclear relaxation times. We find that Fe^3+^ replaces In^3+^ in the Cs_2_AgIn_1−*x*_Fe_*x*_Cl_6_ matrix, easily forming [FeCl_6_]^3−^·[AgCl_6_]^5−^ domains. We also reveal that the sizes and distribution modes of [FeCl_6_]^3−^·[AgCl_6_]^5−^ domains are different in different amounts of Fe^3+^ alloying. Our findings provide fundamental understanding of the atomic-level structure in double perovskite alloys, and are important for rational development of novel alloying elements for optoelectronic applications.

## Results and discussion

**Fig. 1:**
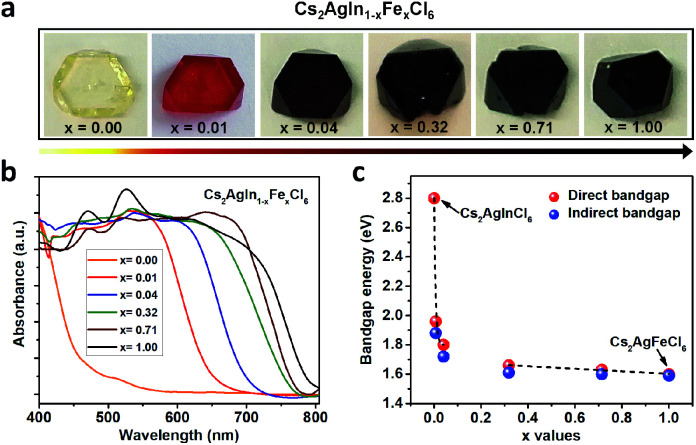
Photographs (a) and normalized UV-Vis absorption spectra (b) of Cs_2_AgIn_1−*x*_Fe_*x*_Cl_6_ (*x* = 0.00, 0.01, 0.04, 0.32, 0.71 and 1.00) crystals. (c) Bandgaps of DPAs Cs_2_AgIn_1−*x*_Fe_*x*_Cl_6_ (*x* = 0.00, 0.01, 0.04, 0.32, 0.71 and 1.00) extracted by linear fits to both direct bandgap and indirect bandgap Tauc plots.

Double perovskite alloys (DPAs) Cs_2_AgIn_1−*x*_Fe_*x*_Cl_6_ (*x* = 0–1) single crystals were synthesized by the hydrothermal method from CsCl, AgCl, InCl_3_, FeCl_3_ and HCl precursor solutions (more details in ESI†). As shown in Fig. 1a, all the resulting crystals exhibit similar truncated octahedral morphology, and the crystal color changes from transparent to black with increasing Fe^3+^ concentration. It is worth noting that the Fe^3+^ can completely substitute In^3+^ in Cs_2_AgInCl_6_ with the formation of Cs_2_AgFeCl_6_, probably due to comparable ion radii between In^3+^ and Fe^3+^.^22^ The accurate concentrations (*x* values) of Fe^3+^ in different crystals are obtained from inductively coupled plasma optical emission spectrometer (ICP-OES) (Table S1†).

In order to understand different crystal colors between DPAs Cs_2_AgIn_1−*x*_Fe_*x*_Cl_6_, we investigate their optical absorption properties through UV-Visible (UV-Vis) reflectance spectra (Fig. S1†), which are further transformed to pseudo-absorption values by the Kubelka–Munk theorem.^23^ As shown in Fig. 1b, there is a nonlinear change in the absorption for these alloys with increasing Fe^3+^ concentration. Specifically, the absorption edges broaden rapidly (from original ∼460 nm to ∼700 nm) with low Fe^3+^-concentrations (*x* ≤ 0.04) (Fig. 1b and S2†), consistent with the observed color changes in Fig. 1a. With increasing Fe^3+^ concentration, the absorption edges of Cs_2_AgIn_1−*x*_Fe_*x*_Cl_6_ slowly broaden. We further determine the optical bandgaps of DPAs Cs_2_AgIn_1−*x*_Fe_*x*_Cl_6_ (*x* = 0.00, 0.01, 0.04, 0.32, 0.71 and 1.00) by plotting *α*^*r*^ as the function of photon energy (*hν*), where *α* is the pseudo-absorption coefficient, the values for *r* are 2 and 1/2 for a direct and indirect bandgap, respectively. Considering the uncertainty of the direct or indirect property of DPAs Cs_2_AgIn_1−*x*_Fe_*x*_Cl_6_ (*x* > 0), we fit the absorption results by both functions (Fig. S3 and S4†). Both analyses indicate that the bandgap decreases quickly at low Fe^3+^-concentrations (*x* ≤ 0.04), followed by a slow decrease at high Fe-concentrations (*x* ≥ 0.32), reaching the smallest bandgap of ∼1.6 eV (Fig. 1c).

**Fig. 2:**
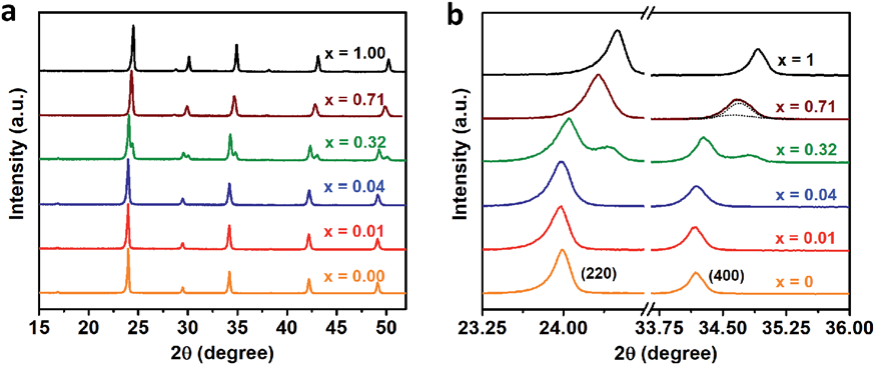
(a) XRD patterns of Cs_2_AgIn_1−*x*_Fe_*x*_Cl_6_ (*x* = 0.00, 0.01, 0.04, 0.32, 0.71 and 1.00) powders with different Fe^3+^-concentrations. (b) The enlarged view of the (220) and (400) diffraction peaks in the PXRD patterns.

We further perform the powder X-ray diffraction (PXRD) measurements to investigate how Fe^3+^-alloying affects the crystal structures of DPAs Cs_2_AgIn_1−*x*_Fe_*x*_Cl_6_. As shown in Fig. 2a, all patterns show a similar diffraction behavior for the entire 2*θ* range, indicating that all DPAs retain the original cubic structure of Cs_2_AgInCl_6_. Interestingly, the change of lattice parameters with increasing concentrations of Fe-alloying can be regarded as two stages (Fig. S5†), in accordance with the absorption results. For low Fe^3+^-concentrations (*x* ≤ 0.04), all the diffraction peaks are almost the same, with no obvious peak shifts (Fig. 2b). Further increasing the Fe^3+^-concentration (*x* ≥ 0.32), the diffraction peaks shift to the high-angle side gradually, which can be understood by the smaller ionic radius of Fe^3+^ (0.65 Å) compared with In^3+^ (0.80 Å). Particularly, both (220) and (400) diffraction peaks split into two peaks for 32% Fe^3+^-alloyed Cs_2_AgInCl_6_, suggesting the segregation of In^3+^-rich and Fe^3+^-rich phases. According to the Scherrer equation, we can estimate the average domain size of In^3+^-rich and Fe^3+^-rich phases are about 37 nm and 24 nm, respectively. These split diffraction peaks tend to merge into a single peak again with further increasing the Fe^3+^ concentration to 71% (Fig. 2b and S6†). In other words, the diffraction peaks split when the Fe^3+^ concentration is comparable with the In^3+^ concentration, implying possible formation of In^3+^-rich and Fe^3+^-rich phases, as confirmed by the following ssNMR measurements.

**Fig. 3:**
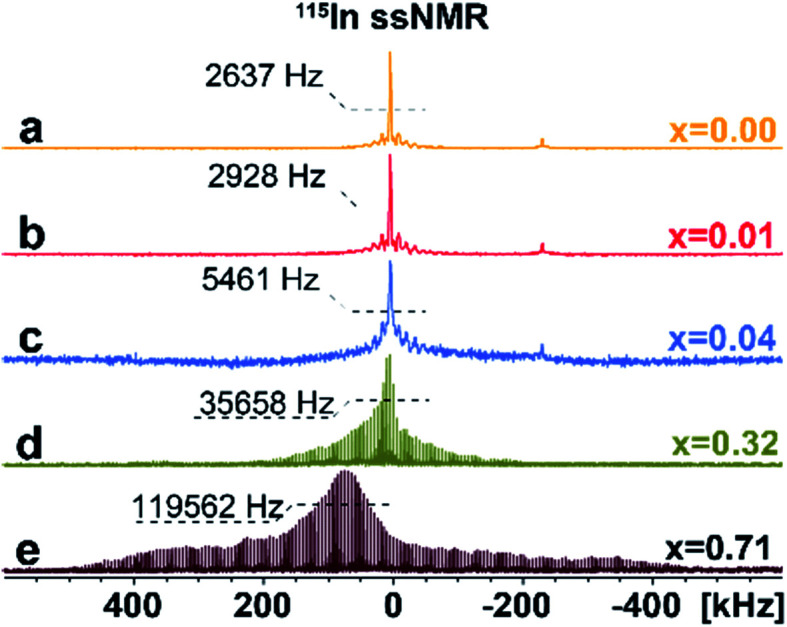
Experimental ^115^In ssNMR spin-echo (a–c) and WURST-QCPMG (d and e) spectra of Cs_2_AgIn_1−*x*_Fe_*x*_Cl_6_ (*x* = 0.00, 0.01, 0.04, 0.32, 0.71), conducted at static conditions. The ^115^In WURST-QCPMG NMR spectra are shown for better clarity. The magnification of ^115^In ssNMR spin-echo spectra (a–c) and comparison of ^115^In ssNMR spectra (spin-echo *vs.* WURST-QCPMG) for (d and e) are listed in ESI, see Fig. S7 and S8,† respectively.

To investigate the distribution of different phases and atomic-level structures, we perform ^133^Cs, ^115^In solid-state NMR (ssNMR) experiments.^16^ As shown in Fig. 3, a relatively narrow, symmetric signal at *ca. δ*_iso_ = 41.6 ppm appears in pristine Cs_2_AgInCl_6_ in ^115^In ssNMR spectra which shifts slightly to lower frequencies with increasing concentrations of Fe. While these signals seem very similar, they contain useful structural information. We observe not only slight changes in the chemical shifts of the detected signal, but also signal broadening, suggesting increasing presence of dopant species (diamagnetic Fe^2+^/paramagnetic Fe^3+^) incorporated in the matrix. The increasing broadening of ^115^In signals with growing amount of Fe dopant species is induced by changes in static chemical disorder (distribution of local environments) as well as second order quadrupolar broadening of the central transition.^16^ In the case of 4% Fe-alloyed perovskite, the resulting ^115^In ssNMR spectrum shows broadened and less resolved spectral line (Fig. 3c and S7†). The increase of alloyed Fe concentration (32% and 71%) highlights this effect as recorded spectra become increasingly broad. This is further documented by increasing signal half-width in ^115^In ssNMR spectra (Fig. 3d and e), induced by the presence of Fe ions in the matrix.

**Fig. 4:**
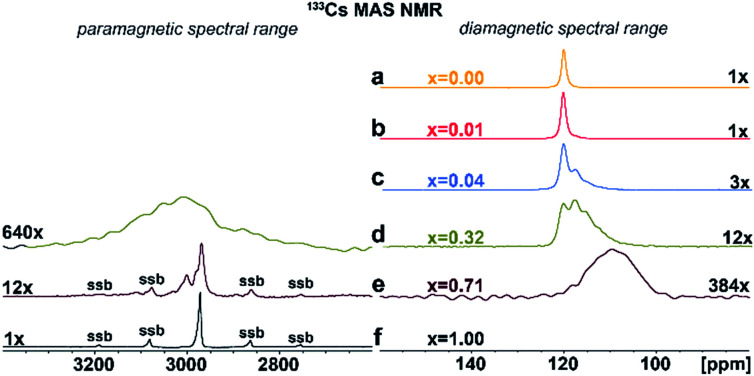
(a)–(f) Experimental ^133^Cs MAS NMR spectra of Cs_2_AgIn_1−*x*_Fe_*x*_Cl_6_ (*x* = 0.00, 0.01, 0.04, 0.32, 0.71 and 1.00). The major detected ^133^Cs NMR signals were confirmed using ^133^Cs–^133^Cs SD/MAS NMR experiments (The ^133^Cs–^133^Cs SD/MAS NMR spectra are listed in ESI, Fig. S9†).

Additional structural information is provided from ^133^Cs NMR spectra. As shown in Fig. 4a–f, the symmetric peak at *δ*_iso_ = 120.2 ± 0.5 ppm for pristine Cs_2_AgInCl_6_ splits, broadens and finally disappears with increasing concentration of Fe ions while at high concentrations a new peak at *δ*_iso_ = 2973 ± 1.0 ppm (Fig. 4d–f) grows. Single, narrow signals for the pristine Cs_2_AgInCl_6_ (Fig. 4a) and the completely substituted Cs_2_AgFeCl_6_ (Fig. 4f) confirm the existence of one crystallographic position of the Cs^+^ ions in both structures. The significant shift of peak from *δ*_iso_ = 120.2 ± 0.5 ppm (Cs_2_AgInCl_6_) to *δ*_iso_ = 2973 ± 1.0 ppm (Cs_2_AgFeCl_6_) in ^133^Cs MAS NMR spectra suggests presence of paramagnetic Fe^3+^ in perovskite matrix. Moreover, with increasing concentration of Fe ions alloyed into Cs_2_AgInCl_6_ lattice, the chemical environment of Cs^+^ becomes more complex as seen by additional signals appearing in the range of 100–120 ppm (Fig. S9†). Specifically, at low Fe concentration (*x* = 0.01), two signals are observed in the ^133^Cs MAS NMR spectra, first remaining at *δ*_iso_ = 120.2 ± 0.5 ppm and a new, less intensive peak appearing at *δ*_iso_ = 117.74 ± 0.5 ppm (Fig. 4b). As the Fe ion concentration increases (*x* ≥ 0.04), the latter signal becomes more significant. For 4% and 32% Fe alloyed systems, a third signal is resolved (Fig. 4c and d), at 111.0 ± 2.0 ppm. The second and third signal (*δ*_iso_ = 117.74 ± 0.5 ppm and 111.0 ± 2.0 ppm) suggest the presence of Fe ions located close to Cs ions. Furthermore, in cases of 32% and 71% Fe alloyed systems, the above mentioned new shifted signal(s) appear at low-frequency position of *ca.* 2950 ± 50 ppm (Fig. 4d and e). These new shifted signal(s) (*δ*_iso_ = 2950 ± 50, 2973 ± 1 and 3002 ± 1 ppm) seem to correspond to the splitting of peaks in the PXRD patterns (Fig. 2b and S6†). In addition, the small changes of ^133^Cs chemical shift in the diamagnetic region (ranging from 200 to (−100) ppm) can be explained by two ways: (i) through-space (pseudo-contact) interactions with paramagnetic Fe^3+^.^24–26^ and/or (ii) the diamagnetic effect of substitution and lattice contraction.^16^ The signals in the diamagnetic region as well as in paramagnetic spectral range around 2950 ppm, indicate multi-component nature of the DPAs, agreeing well with the PXRD results. Based on ^115^In and ^133^Cs ssNMR results, we conclude that the Fe^3+^ ions are incorporated into the matrix replacing In^3+^ ions.

To confirm the presence of paramagnetic Fe^3+^ ions and provide further information on their distribution in DPAs matrix, we perform ^133^Cs NMR *T*_1_-relaxation measurements.^27,28^ These are based on the fact that the presence of paramagnetic metal centers (*e.g.* Fe^3+^) usually causes extremely rapid longitudinal and transverse relaxation of the nearby nuclei due to electron-spin couplings. This approach was developed by Emsley and co-workers and it is based on the reduction of ^1^H and ^133^Cs NMR *T*_1_-relaxation times to detect paramagnetic dopants in lead halide perovskites.^27,28^ The saturation-recovery build–up curves of the detected ^133^Cs NMR signal(s) are analyzed by single and/or multi-exponential functions and values of *T*_1_ (^133^Cs) relaxation times as well as the corresponding fractions of individual components are obtained, see Table 1. Corresponding well with the symmetric single peaks of the Cs_2_AgInCl_6_ and Cs_2_AgFeCl_6_ systems in ^133^Cs MAS NMR spectra, the relaxation decays were fitted by a single exponential function. However, both systems provide significantly different relaxation times with a long *T*_1_ (^133^Cs) relaxation time of 100 s for Cs_2_AgInCl_6_ and a very short *T*_1_ (^133^Cs) relaxation time of 11 ms for Cs_2_AgFeCl_6_. These results further confirm one crystallographic position of the Cs^+^ ions in both systems in an altered unit cell.

**Table 1:** ^133^Cs *T*_1_ relaxation times of Cs_2_AgIn_1−*x*_Fe_*x*_Cl_6_ (*x* = 0.00, 0.01, 0.04, 0.32, 0.71 and 1.00)

Materials	^133^Cs *δ*_iso_ (ppm)	^133^Cs *T*_1_^a^ (s ms^−1^)	Rel. amount (%)
Cs_2_AgInCl_6_	120.2 ± 0.5	101 s	100
Cs_2_AgIn_0.99_Fe_0.01_Cl_6_	120.2 ± 0.5	7.7 s	60 ± 10
	117.7 ± 0.5	1.4 s	40 ± 10
Cs_2_AgIn_0.96_Fe_0.04_Cl_6_	120.2 ± 0.5	420 ms	50 ± 10
	117.7 ± 0.5	63 ms	25 ± 10
	111.0 ± 2.0	58 ms	25 ± 10
Cs_2_AgIn_0.68_Fe_0.32_Cl_6_	120.2 ± 0.5	119 ms	30 ± 10
	117.7 ± 0.5	56 ms	40 ± 10
	111.0 ± 2.0	34 ms	30 ± 10
	2950 ± 50^b^	—	<5 ± 10
Cs_2_AgIn_0.29_Fe_0.71_Cl_6_	111.0 ± 2.0	1.2 s	10 ± 10
	2973 ± 1.0	8.4 ms	60 ± 10
	3002 ± 1.0	7.2 ms	30 ± 10
Cs_2_AgFeCl_6_	2973 ± 1.0	11 ms	100

a The plots of the ^133^Cs *T*_1_ relaxation datasets with the fitted curves are depicted in ESI, see Fig. S10.

b The ^133^Cs *T*_1_ relaxation time cannot be determined due to low concentration and poor resolution of 1D ^133^Cs MAS NMR spectrum (see Fig. 4d).

Contrary to the pristine materials, alloyed Cs_2_AgIn_1−*x*_Fe_*x*_Cl_6_ systems exhibit multi-exponential behavior reflected by a dispersion of observed *T*_1_ (^133^Cs) relaxation times corresponding to distinct distributions of paramagnetic species in the matrix. The 1% Fe^3+^-alloyed Cs_2_AgInCl_6_ system is characterized by a double exponential decay with *T*_1_ (^133^Cs) relaxation times of 7.7 s (60%) and 1.4 s (40%), confirming a two-component character of the matrix. The rapid-relaxation (1.4 s) and the slow-relaxation phases (7.7 s) correspond to a phase extensively occupied by well-dispersed Fe^3+^ ions and the presence of Cs^+^ ions more distant from Fe^3+^ species, respectively. It is noted that the observed significant shortening of both *T*_1_ (^133^Cs) spin-lattice relaxation times as compared to the Cs_2_AgInCl_6_ parent system (100 s) indicates almost homogeneous distribution of Fe^3+^ ions (isolated [FeCl_6_]^3−^ octahedrons and/or small [FeCl_6_]^3−^·[AgCl_6_]^5−^ domains in size up to two unit cells) in the perovskite matrix. With increasing amount of alloyed Fe^3+^ ions (4% and 32%) in the matrix, the multi-component relaxation is further accelerated. The relaxation process is in range of tens and/or hundreds of milliseconds, representing ^133^Cs species in strong interaction with the dispersed Fe^3+^ ions. The significant shortening of relaxation times indicates presence of larger [FeCl_6_]^3−^·[AgCl_6_]^5−^ domains, which mean that the [FeCl_6_]^3−^·[AgCl_6_]^5−^ domains grow larger as the Fe^3+^-concentration increases.

Moreover, for the 4% Fe^3+^ alloyed perovskite, no signal at position 2950 ± 50 ppm was detected and a relatively long *T*_1_ relaxation time (420 ms) for signal at 120.2 ± 0.5 ppm was observed, which suggests the existence of large, homogeneously distributed [FeCl_6_]^3−^·[AgCl_6_]^5−^ domains in Cs_2_AgInCl_6_ parent matrix. In contrast, a new and broad signal at 2950 ± 50 ppm observed for 32% Fe^3+^ alloyed perovskite system, points to formation of a secondary Fe^3+^-rich phase, also confirmed by observed very short relaxation times (120–30 ms). Besides, the visible broadening of the signal at 2950 ± 50 ppm indicates the presence of static disorder which implies random distribution of these [FeCl_6_]^3−^·[AgCl_6_]^5−^ domains in the matrix. Combining the short relaxation times and additional peaks in ^133^Cs MAS NMR spectra (Table 1 and Fig. 4d), we can conclude that for 32% Fe^3+^ alloyed perovskite system the [FeCl_6_]^3−^·[AgCl_6_]^5−^ domains have grown to form a second, interconnected microscopic phase (Fe^3+^-rich phase). These results correspond well with the feature in PXRD data, caused mainly by interconnection between these larger [FeCl_6_]^3−^·[AgCl_6_]^5−^ domains.

In case of 71% Fe^3+^ alloyed system, we also conclude a two-component system based on ^133^Cs MAS NMR spectra (Fig. 4e) and *T*_1_ (^133^Cs) spin-lattice relaxation times. In ^133^Cs MAS NMR spectra, two relatively well-ordered phases of Cs_2_AgFeCl_6_ are confirmed by two sharp signals at 2973 and 3002 ppm while the [InCl_6_]^3−^·[AgCl_6_]^5−^ domains are represented by the signal at 111.0 ± 2.0 ppm. The matrix can be defined as an inverted-phase system as compared to the above-mentioned systems. The distribution of [InCl_6_]^3−^·[AgCl_6_]^5−^ domains can also be derived from the unexpectedly relatively long ^133^Cs *T*_1_ relaxation time (1.2 s, see Table 1.) of the corresponding signal. Considering that similarly long relaxation time was detected for the second phase of the 1% Fe alloyed Cs_2_AgInCl_6_, we conclude that the formation of relatively large [InCl_6_]^3−^·[AgCl_6_]^5−^ domains surrounded by [FeCl_6_]^3−^ matrix. Within these domains some substitution by Fe^3+^ ions can be presumed based on the value of the corresponding relaxation time. On the other hand, the very short relaxation times for the peaks at 2973 ± 1.0 and 3002 ± 1.0 ppm confirm formation of large [FeCl_6_]^3−^·[AgCl_6_]^5−^ domains with slightly different local environments.

**Fig. 5:**
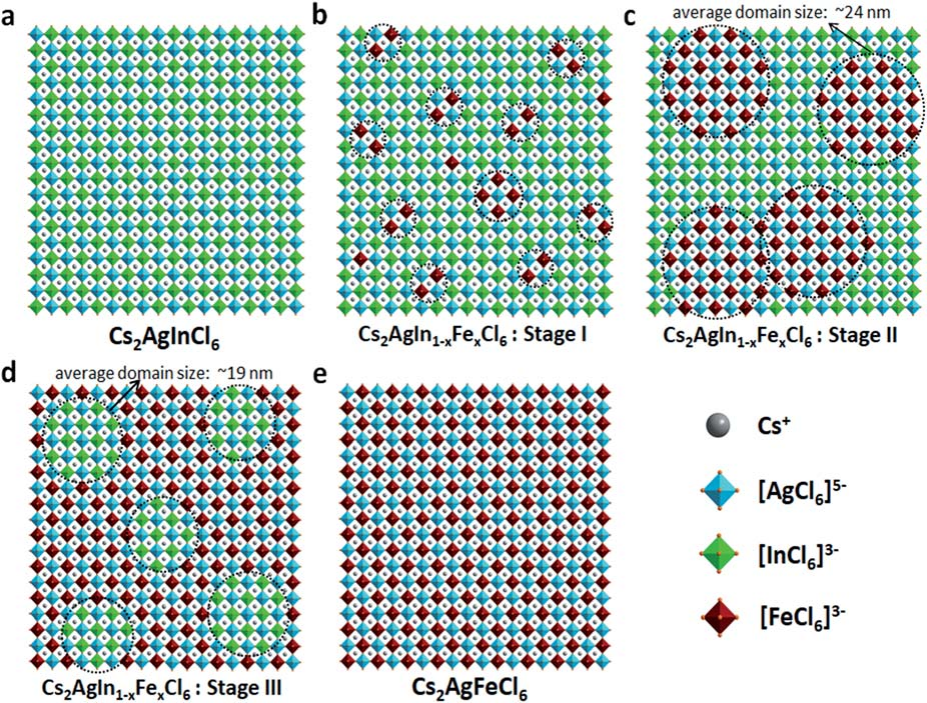
(a)–(e) Schematic presentations of possible scenarios for Fe^3+^ distribution inside the DPAs Cs_2_AgIn_1−*x*_Fe_*x*_Cl_6_ (*x* = 0–1) matrix. The [FeCl_6_]^3−^·[AgCl_6_]^5−^ or [InCl_6_]^3−^·[AgCl_6_]^5−^ domains are highlighted in balck circles.

In short, based on the above observations, both pure Cs_2_AgInCl_6_ and Cs_2_AgFeCl_6_ are homogeneous systems (Fig. 5a and e). Meanwhile, the Fe-alloying process in DPAs Cs_2_AgIn_1−*x*_Fe_*x*_Cl_6_ can be divided into the following three stages with different Fe^3+^ concentrations:

(i) For low Fe^3+^ concentration DPAs (*x* ≤ 0.04), Fe^3+^ ions exist in the form of isolated [FeCl_6_]^3−^ octahedrons and/or small [FeCl_6_]^3−^·[AgCl_6_]^5−^ domains, which are almost homogeneously distributed in the perovskite matrix (Fig. 5b, stage I).

(ii) For medium Fe^3+^ concentration DPAs, the isolated [FeCl_6_]^3−^ octahedrons or small [FeCl_6_]^3−^·[AgCl_6_]^5−^ domains grow into larger [FeCl_6_]^3−^·[AgCl_6_]^5−^ domains. The growth of these domains forms microscopically segregated phases with different sizes, leading to nonhomogeneous distribution in the matrix (Fig. 5c, stage II). A typical example is DPAs Cs_2_AgIn_1−*x*_Fe_*x*_Cl_6_ with 32% Fe^3+^ concentration. The average domain size of [FeCl_6_]^3−^·[AgCl_6_]^5−^ domains in the 32% Fe^3+^ alloyed sample is about 24 nm.

(iii) For high Fe^3+^ concentration DPAs (*x* ≥ 0.71), the structure can be viewed as low concentration In^3+^-alloyed Cs_2_AgFeCl_6_. In this case, DPAs become a relatively uniform phase with almost homogeneous distribution of small and/or relatively large [InCl_6_]^3−^·[AgCl_6_]^5−^ domains in the Cs_2_AgFeCl_6_ matrix (Fig. 5d, stage III).

## Conclusions

In conclusion, we successfully tune the bandgap of Cs_2_AgInCl_6_ from 2.8 eV to 1.6 eV through Fe^3+^-alloying, which is attractive for optoelectronic device applications. Moreover, we provide fundamental understanding of the atomic-level structure of DPAs with paramagnetic alloying ions (Cs_2_AgIn_1−*x*_Fe_*x*_Cl_6_), as revealed by the ^133^Cs/^115^In ssNMR spectroscopy. Our results indicate that paramagnetic Fe^3+^ replaces diamagnetic In^3+^ in Cs_2_AgIn_1−*x*_Fe_*x*_Cl_6_ matrix and forms [FeCl_6_]^3−^·[AgCl_6_]^5−^ domains, which grow larger as the Fe^3+^ concentration increases. Meanwhile, the connection of these larger [FeCl_6_]^3−^·[AgCl_6_]^5−^ domains leads to the formation of microscopically segregated Fe^3+^-rich phases in DPAs. We believe that ssNMR is also widely suitable for atomic-level structure study in traditional magnetic semiconductors (*e.g.* GaAs : Mn), molecular magnets, *etc.*

## Conflicts of interest

There are no conflicts to declare.

## Supplementary Material

SC-012-D0SC05264G-s001
