# Supplementary material for: The atomic-level structure of bandgap engineered double perovskite alloys Cs2AgIn1−xFexCl6
Source: Chem Sci. 2020 Dec 8;12(5):1730–5. doi: 10.1039/d0sc05264g (PMC8179106; doi:10.1039/d0sc05264g)
Supplement: SC-012-D0SC05264G-s001 [file SC-012-D0SC05264G-s001.pdf]

Electronic Supplementary Material (ESI) for Chemical Science.
This journal is © The Royal Society of Chemistry 2020

Supporting Information

The Atomic-Level Structure of Bandgap Engineered Double Perovskite Alloys $Cs_2AgIn_xFe_{1-x}Cl_6$

Fuxiang Ji,[a] Feng Wang,[a] Libor Kobera,[b] Sabina Abbrent,[b] Jiri Brus,[b] Weihua Ning*[ac] and Feng Gao*[a]*

[a] Department of Physics, Chemistry and Biology (IFM), Linköping University, Linköping SE-581 83, Sweden

[b] Institute of Macromolecular Chemistry of the Czech Academy of Sciences, Heyrovskeho nam. 2, 162 06, Prague 6, Czech Republic

[c] Key Laboratory of Flexible Electronics (KLOFE) and Institute of Advanced Materials (IAM), Nanjing Tech University, 30 South Puzhu Road, Nanjing 211816, P. R. China

EXPERIMENTAL SECTION

Synthesis. All the chemicals used were purchased from Sigma-Aldrich without any further purification. For pristine $Cs_2AgInCl_6$ crystals, solid CsCl (168.4 mg, 1.00 mmol), $InCl_3$ (110.6 mg, 0.5 mmol) and AgCl (71.7 mg, 0.5 mmol) were dissolved in 7 mL of 37% HCl and then transfer into a 25 cm^3 Teflon-lined autoclave. The autoclave was sealed and placed in the oven where it was heated to 180 ^{o}C for 12 h. After being slowly cooled to room temperature at a rate of 1 $^{o}C/hr$, transparent octahedral $Cs_2AgInCl_6$ single crystals were achieved. For Fe-alloyed $Cs_2AgInCl_6$ crystals, x% (molar ratio) $InCl_3$ is replaced by equimolar $FeCl_3$ with 1%, 20%, 50%, 80%, 100% in the precursor solutions, respectively. The synthesis approach is the same as that used for pristine $Cs_2AgInCl_6$.

Physical measurements. The XRD patterns of the products were recorded with a X'Pert PRO X-ray diffractometer using Cu $K\alpha_1$ irradiation (λ = 1.5406 Å). The Ultraviolet–Visible reflectance spectra were measured with a PerkinElmer model Lambda 900. Inductively coupled plasma optical emission spectrometer (ICP-OES) analysis of $Cs_2AgIn_xFe_{1-x}Cl_6$ samples was performed by Agilent 5110. We measured In^{3+} and Fe^{3+} concentration in $Cs_2AgIn_xFe_{1-x}Cl_6$ solution dissolved by HCl. This data was used to compute the In^{3+}/Fe^{3+} ratio of $Cs_2AgIn_xFe_{1-x}Cl_6$.

Solid state Nuclear Magnetic Resonance analysis. Solid-state NMR spectra (ssNMR) were recorded at 11.7 T using a Bruker AVANCE III HD spectrometer. The 4 mm cross-polarization magic angle spinning (CP/MAS) probe was used for ^{133}Cs and ^{115}In experiments recorded at Larmor frequency of $\nu(^{133}Cs)$ = 65.611 MHz and $\nu(^{115}In)$ =

109.950 MHz, respectively. ^{133}Cs MAS NMR (single-pulse) experiments were collected at 7 kHz spinning speed with ^{1}H decoupling (SPINAL 64). The recycle delay was 4 s for all ssNMR experiments with used number of scans 4-1024. The ^{133}Cs chemical shift was calibrated using solid CsCl (^{133}Cs: 228.1 ppm).[1] The pulse length was set to 2.4 μs at 100 W for maximal signal intensity. The ^{133}Cs-^{133}Cs correlation MAS NMR spectra were recorded using NOESY-type three-pulse sequence. Spectral width in both frequency dimensions was 14 kHz. The indirect detection period t_1 consisted of 128 increments each made of 48 scans. The ^{133}Cs spin-lattice relaxation experiments were measured using the standard saturation-recovery experiment. For initial saturation of ^{133}Cs spins the train of 200 pulses separated by a 0.005 s delay was used. The applied variable delays covered the time region from 10 μs to 100 s. The experiment was performed at spinning frequency of 7 kHz and 24 of number of scans for each increment. The ^{115}In chemical shift was calibrated using a saturated solution of 0.1M $In(NO_3)_3$ in diluted HNO_3 (^{115}In: 0.0 ppm).[2] The CT-selective $\pi/2$ pulse length was 1.26 μs at 40 W. ^{115}In NMR experiments were collected at static conditions using spin-echo NMR experiments (90°-τ-180°-acq.),[3] the delay between pulses was 160 μs. The ^{115}In WURST-QCPMG NMR experiment was carried out using a 50 μs CT-selective WURST pulse using 1 MHz sweep width, with 64 loops and step 300 kHz. The recycle delay was 2 s for all ^{115}In ssNMR spectra.

To compensate for frictional heating of the spinning samples, all NMR experiments were measured under active cooling. Sample temperature was maintained at 298 K and temperature calibration was performed on $Pb(NO_3)_2$ using a calibration procedure

described in the literature.[4] Dried samples were packed into ZrO_2 rotors and subsequently stored at room temperature. All NMR spectra were processed using Top Spin 3.5 pl2 software package.

Optical band-gap determination. The reflectance spectra we obtained were converted to pseudo-absorbance spectra using the Kubelka-Munk transform.

$\alpha \approx (1\text{-}R)^2/2R$

where α = pseudo-absorbance and R = reflection. The direct and indirect bandgaps were measured by taking the intercept upon extrapolation of the linear regions of $(\alpha hv)^2$ Vs E(eV) and $(\alpha hv)^{1/2}$ Vs E(eV) plots, respectively.

Table S1. Inductively coupled plasma optical emission spectrometer (ICP-OES) results of $Cs_2AgIn_{1-x}Fe_xCl_6$

Sample Labels (in the precursors)	Fe% (Mass percentage)	In% (Mass percentage)	ICP-OES results (in the crystals)
$In_{0.99}Fe_{0.01}$	0.08	24.31	$In_{0.993}Fe_{0.007}$
$In_{0.8}Fe_{0.2}$	0.38	18.61	$In_{0.96}Fe_{0.04}$
$In_{0.5}Fe_{0.5}$	4.21	18.54	$In_{0.672}Fe_{0.318}$
$In_{0.2}Fe_{0.8}$	6.44	5.3	$In_{0.286}Fe_{0.714}$

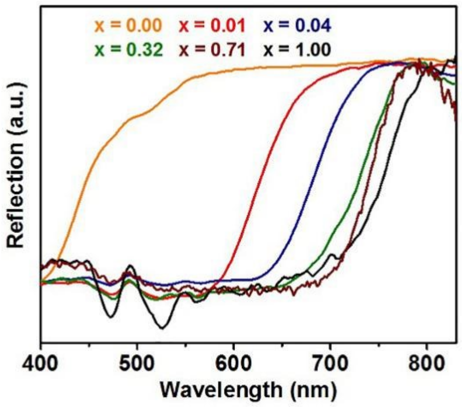

Figure S1. Normalized UV-Vis reflectance spectra of $Cs_2AgIn_{1-x}Fe_xCl_6$ (x = 0.00, 0.01, 0.04, 0.32, 0.71 and 1.00).

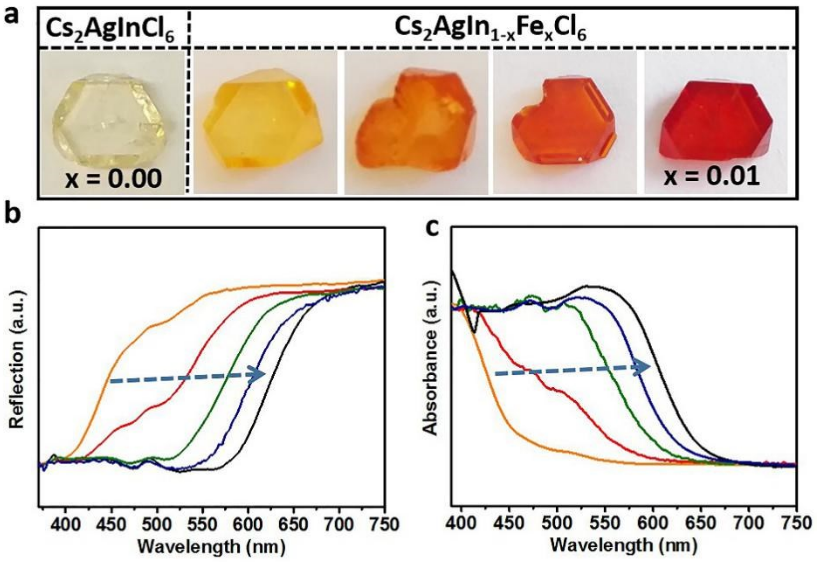

Figure S2. (a) Photograph of single crystals of $Cs_2AgIn_{1-x}Fe_xCl_6$ ($0 \leq x \leq 0.01$). Normalized UV-Vis reflection (b) and absorption spectra (c) of $Cs_2AgIn_{1-x}Fe_xCl_6$ ($0 \leq x \leq 0.01$).

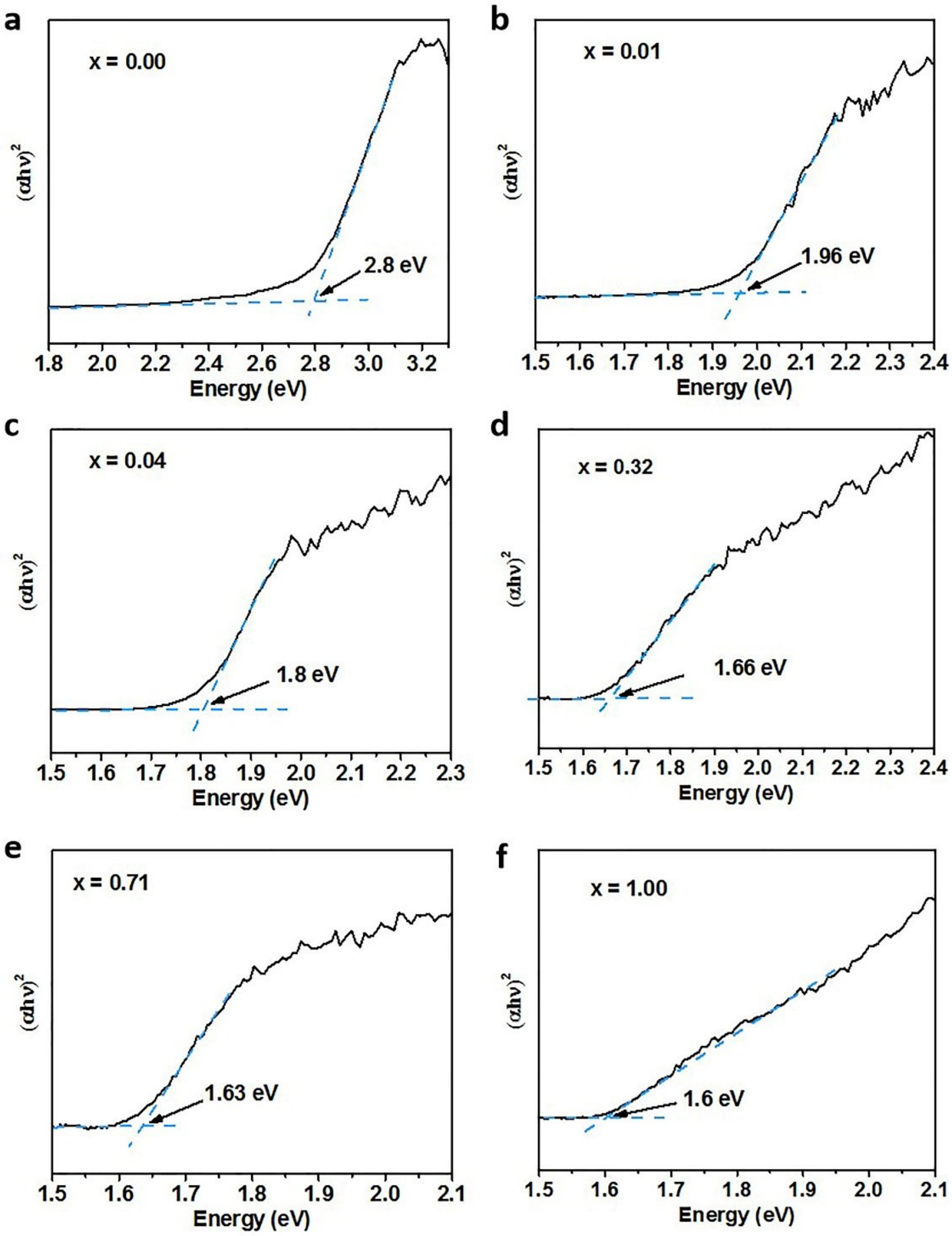

Figure S3. Plots of α^2 versus E extracted from UV-Vis reflectance spectra of $Cs_2AgIn_{1-x}Fe_xCl_6$ crystals of (a) x=0.00, (b) x=0.01, (c) x=0.04, (d) x=0.32, (e) x=0.71 and (f) x=1.00 for an assumed direct bandgap, showing the linear fits.

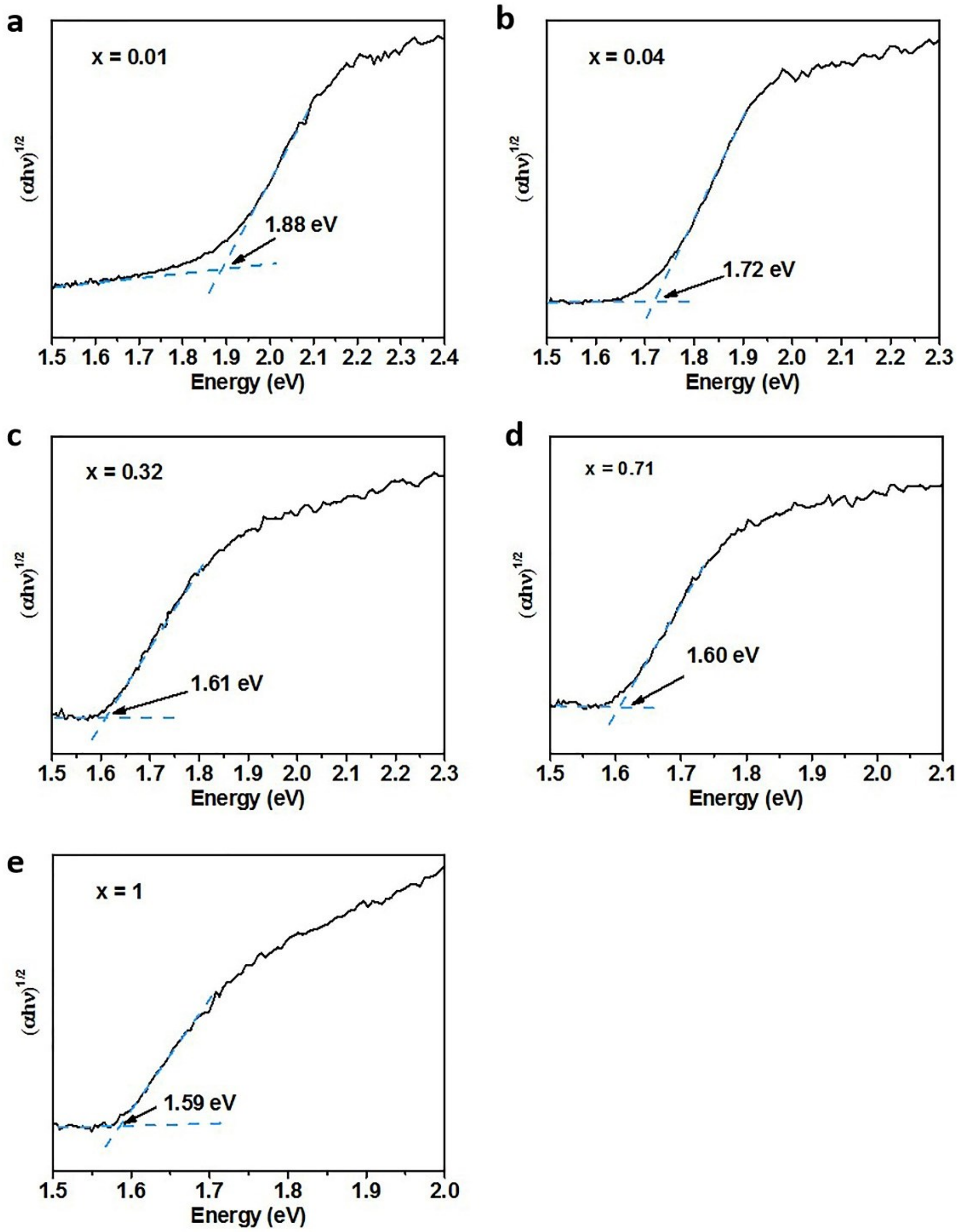

Figure S4. Plots of $\alpha^{1/2}$ versus E extracted from UV-Vis reflectance spectra of $Cs_2AgIn_{1-x}Fe_xCl_6$ crystals of (a) x=0.01, (b) x=0.04, (c) x=0.32, (d) x=0.71 and (e) x=1.00 for an assumed indirect bandgap, showing the linear fits.

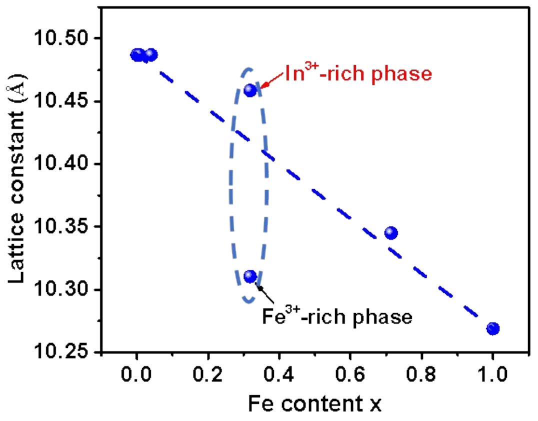

Figure S5. The variation of cubic crystal lattice parameter a as a function of substitution level x. The lattice parameters are calculated based on the (220) diffraction peak.

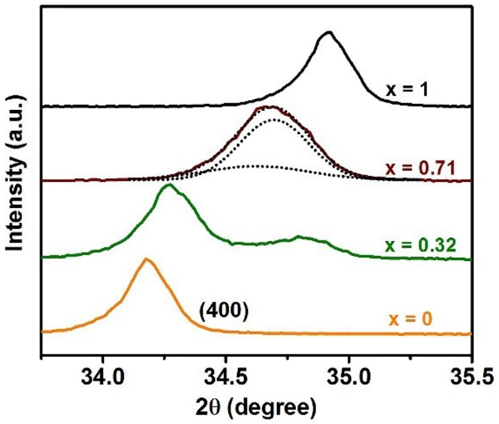

Figure S6. The enlarged view of the (400) diffraction peaks in the PXRD patterns.

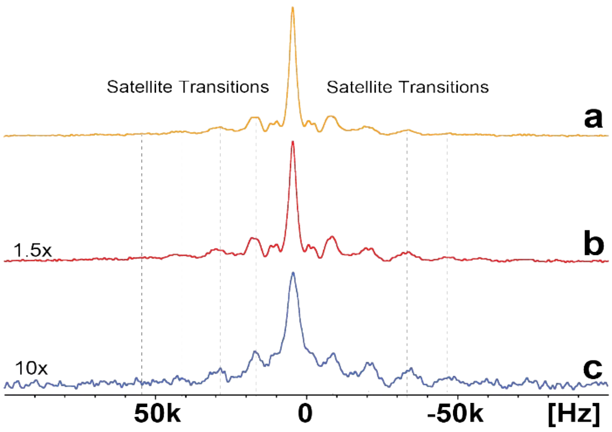

Figure S7. The magnification of experimental ^{115}In ssNMR spin-echo spectra (a-c) in Figure 3.

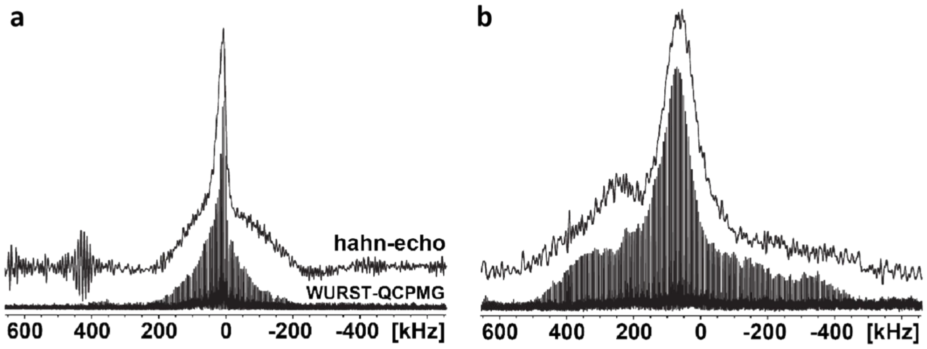

Figure S8. The comparison of experimental ^{115}In ssNMR spectra (spin-echo vs WURST-QCPMG) for $Cs_2AgIn_{0.68}Fe_{0.32}Cl_6$ (a) and $Cs_2AgIn_{0.29}Fe_{0.71}Cl_6$ (b).

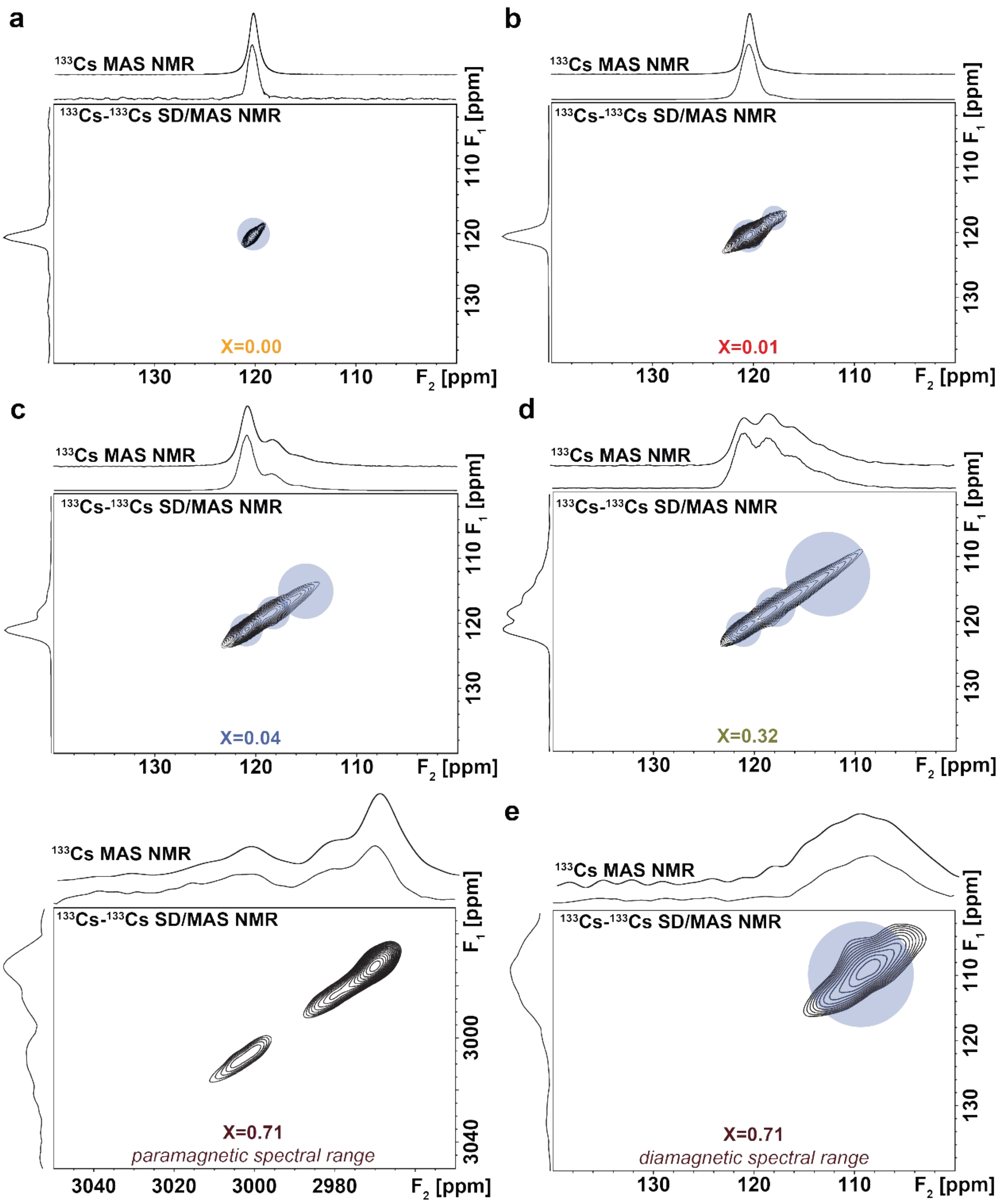

Figure S9. Experimental ^{133}Cs-^{133}Cs SD/MAS NMR and ^{133}Cs MAS NMR spectra of $Cs_2AgIn_{1-x}Fe_xCl_6$ (x = 0.00, 0.01, 0.04, 0.32, 0.71). The spectra were recorded at very short spin diffusion mixing time (10 μs) to enhance spectral resolution of diagonal signals.

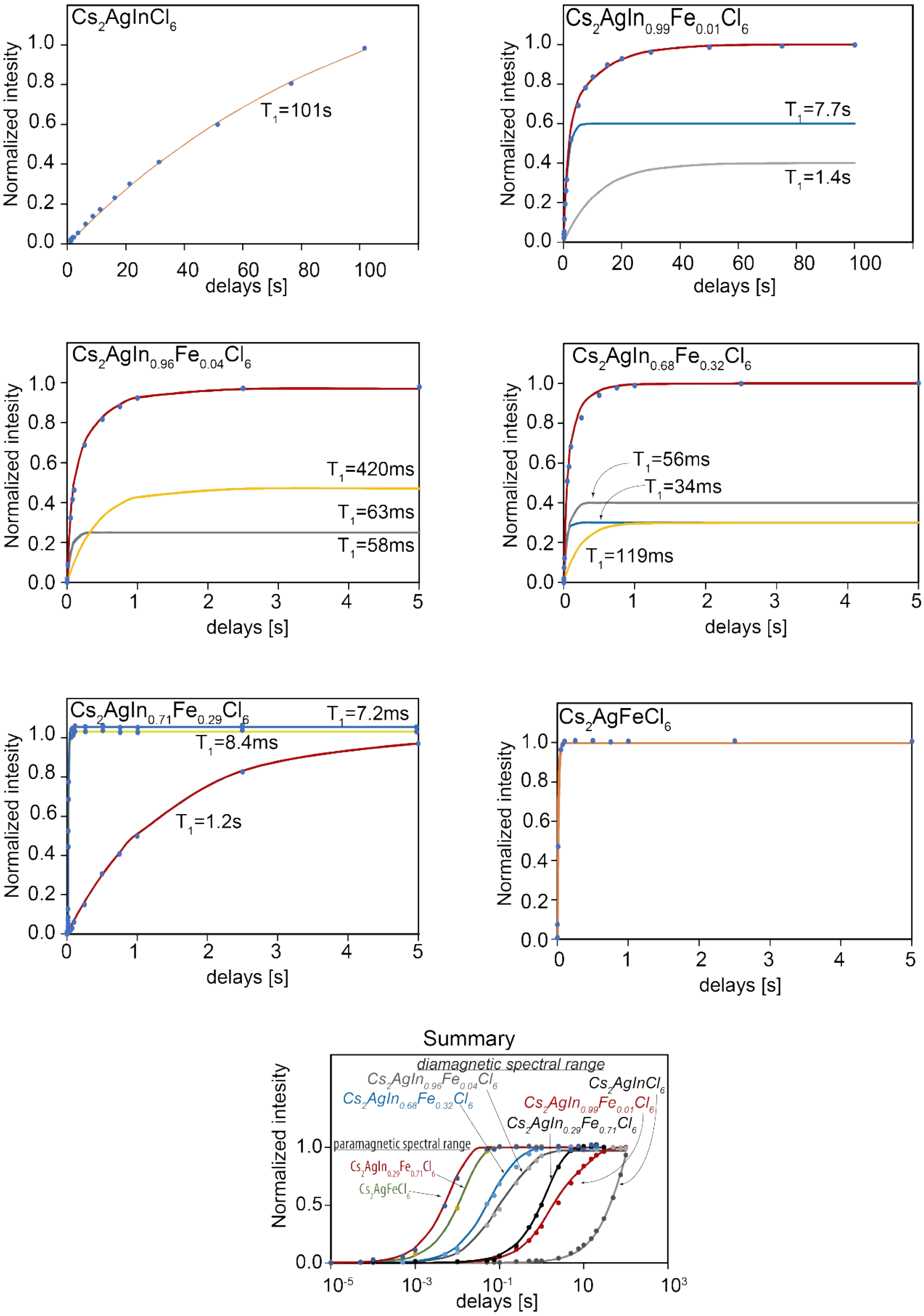

Figure S10. The plots of the [133]Cs T_1 relaxation datasets with the fitted curves spectra of $Cs_2AgIn_{1-x}Fe_xCl_6$ (x = 0.00, 0.01, 0.04, 0.32, 0.71 and 1).

References

1 A. R. Haase, M. A. Kerber, D. Kessler, J. Kronenbitter, H. Krüger, 0. Lutz, M. Müller and A. Nolle, *Z. Naturforsch.*, 1977, **32 a**, 952–956.

2 F. Chen, G. Ma, G. M. Bernard, R. G. Cavell, R. McDonald, M. J. Ferguson and R. E. Wasylishen, *J. Am. Chem. Soc.*, 2010, **132**, 5479–5493.

3 E. L. Hahn, *Phys. Rev.*, 1950, **80**, 580–594.

4 J. Brus, *Solid State Nuclear Magnetic Resonance*, 2000, **16**, 151–160.
